# Zinc supplementation alters airway inflammation and airway hyperresponsiveness to a common allergen

**DOI:** 10.1186/1476-9255-8-36

**Published:** 2011-12-07

**Authors:** Carrie I Morgan, John R Ledford, Ping Zhou, Kristen Page

**Affiliations:** 1 Department of Pediatrics, Cincinnati Children's Hospital Medical Center, Cincinnati, OH, USA; 2 Department of Pediatrics, University of Cincinnati, Cincinnati, OH, USA

## Abstract

**Background:**

Zinc supplementation can modulate immunity through inhibition of NF-κB, a transcription factor that controls many immune response genes. Thus, we sought to examine the mechanism by which zinc supplementation tempers the response to a common allergen and determine its effect on allergic airway inflammation.

**Methods:**

Mice were injected with zinc gluconate prior to German cockroach (GC) feces (frass) exposure and airway inflammation was assessed. Primary bone marrow-derived neutrophils and DMSO-differentiated HL-60 cells were used to assess the role of zinc gluconate on tumor necrosis factor (TNF)α expression. NF-κB:DNA binding and IKK activity were assessed by EMSA and *in vitro *kinase assay. Protein levels of A20, RIP1 and TRAF6 were assessed by Western blot analysis. Establishment of allergic airway inflammation with GC frass was followed by administration of zinc gluconate. Airway hyperresponsiveness, serum IgE levels, eosinophilia and Th2 cytokine production were assessed.

**Results:**

Administration of zinc gluconate prior to allergen exposure resulted in significantly decreased neutrophil infiltration and TNFα cytokine release into the airways. This correlated with decreased NF-κB activity in the whole lung. Treatment with zinc gluconate significantly decreased GC frass-mediated TNFα production from bone-marrow derived neutrophils and HL-60 cells. We confirmed zinc-mediated decreases in NF-κB:DNA binding and IKK activity in HL-60 cells. A20, a natural inhibitor of NF-κB and a zinc-fingered protein, is a potential target of zinc. Zinc treatment did not alter A20 levels in the short term, but resulted in the degradation of RIP1, an important upstream activator of IKK. TRAF6 protein levels were unaffected. To determine the application for zinc as a therapeutic for asthma, we administered zinc following the establishment of allergic airway inflammation in a murine model. Zinc supplementation decreased airway hyperresponsiveness and serum IgE levels, but had no effect on Th2 cytokine expression.

**Conclusions:**

This report suggests that the mechanism by which zinc supplementation alters NF-κB activity is via the alteration of A20 activity. In addition, this study provides evidence that supplementation of zinc to asthmatics may alter airway reactivity and serum IgE levels, suggesting zinc supplementation as a potential treatment for asthmatics.

## Introduction

Zinc is an essential trace element acquired by dietary means. It plays a central role in modulating the immune system and is essential for cellular function in the immune response as well as acting as an antioxidant [1]. The syndrome of zinc deficiency in humans is characterized by susceptibility to infections and in a mouse model of polymicrobial sepsis, zinc deficiency increased organ damage and mortality [2]. Zinc is known to modulate the immune system via the NF-κB pathway, a ubiquitous transcription factor that controls many immune response genes including cytoplasmic cytokines, decreasing the inflammatory response; however the mechanism by which this occurs is currently unclear [3]. A20 is an important regulator protein of NF-κB, acting as an inhibitor to shut down its activation. Its transcription is induced by NF-κB, suggesting a negative feedback loop to temper inflammation [4]. A20 is also a seven zinc finger protein that functions as an ubiquitin-editing enzyme downstream in the TNFR (tumor necrosis factor receptor 1) and TLR (toll-like receptor) pathways toward NF-κB activation. In order to inhibit NF-κB activation, A20 alters the ubiquitination of receptor-interacting protein 1(RIP1), a protein crucial for the activation of IKK, to target it for degradation and also inactivates TNF receptor-associated factor 6 (TRAF6) by inhibiting its polyubiquitination [5]. Thus A20, as a critical player in suppressing NF-κB activation [6] and as a zinc-containing protein, became a potential target for the location of zinc's effect in immune modulation.

Because of its immune modulating effects, zinc has also gained considerable interest with respect to airway inflammation and asthma. Many articles have documented a relative zinc deficiency in asthmatics, or patients who wheeze, implicating a loss of inflammatory modulation as a potential initiator of symptoms. Serum zinc levels in asthmatics have been found to be lower when compared to non-asthmatics [7,8]; hair zinc levels were lower in wheezy infants when compared to healthy controls [9]; and finally, zinc levels in the sputum of asthmatics were significantly lower than in healthy subjects and were associated with increased frequency of wheeze, severity of asthma, and worse lung function [10]. With this data in mind, and with the ability to supplement zinc in an already established model of airway inflammation, we also sought to examine zinc's effect on airway inflammation and reactivity with the hope that it may be considered as a therapeutic medication in relevant patients.

In these studies, we were able to reaffirm zinc's action as an anti-inflammatory agent via inhibition of the NF-κB pathway and present evidence that zinc alters the activity of inhibitory protein A20. In addition, our data support a role for zinc supplementation in decreasing airway reactivity and serum IgE levels in a murine model of experimental allergic airway inflammation.

## Materials and methods

### GC frass

Fecal remnants (frass) were collected from German cockroaches (*Blattella germanica*) and reconstituted as previously described in endotoxin-free PBS [11]. The frass preparation was frozen in aliquots and the same batch was used throughout the experiments.

### Animals and GC frass exposure

**Figure 1:**
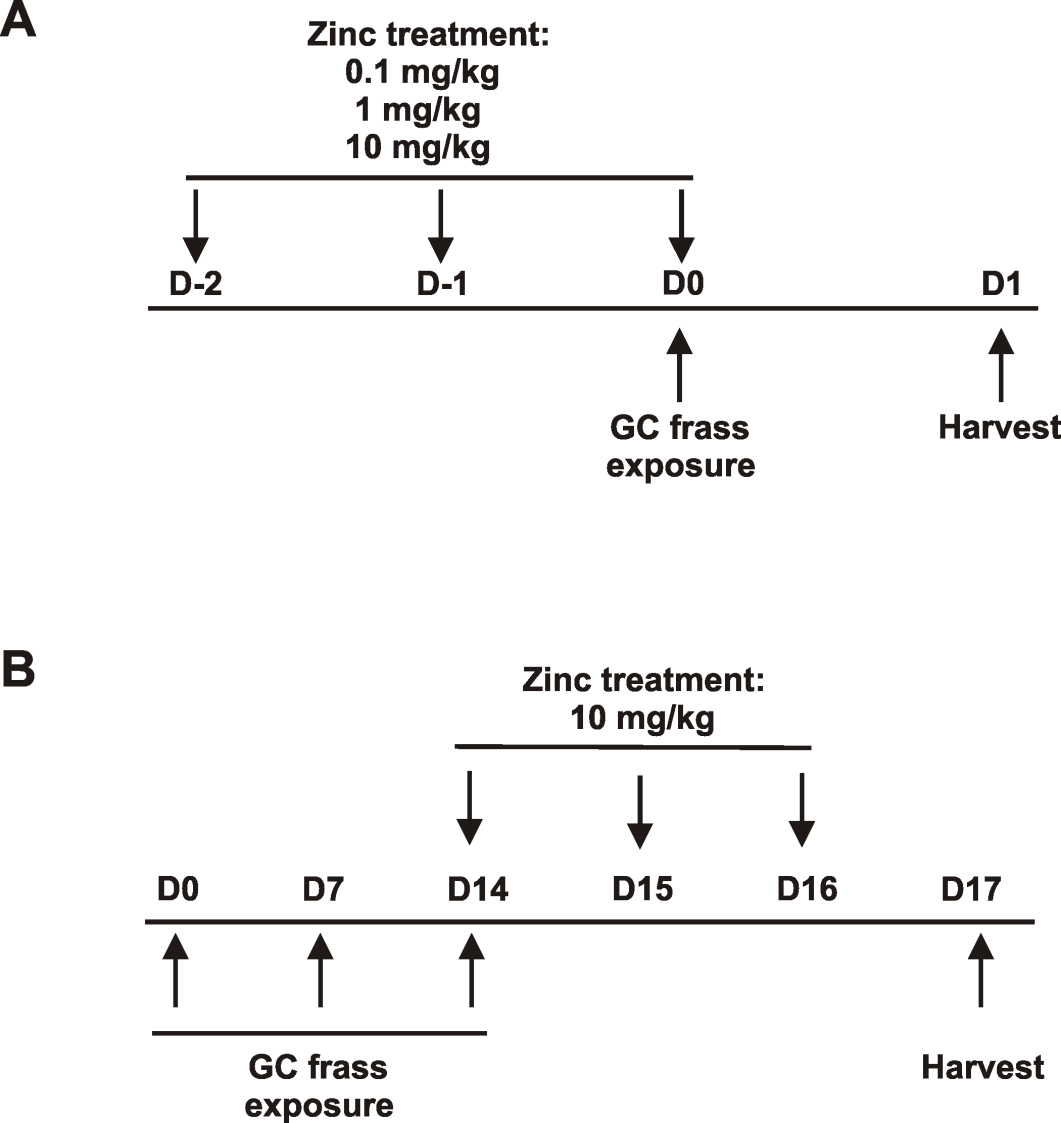
**Mouse treatment protocols**. A. Zinc supplementation followed by GC frass exposure. B. Allergen challenge with GC frass followed by zinc supplementation.

Six week old female BALB/c mice were obtained from Jackson Laboratory (Bar Harbor, ME). Administration of zinc gluconate (0.1, 1 and 10 mg/kg) was performed by intraperitoneal (i.p.) injection for 3 days. On the third day, mice (n = 6 mice/group) were anesthetized with ketamine (45 mg/kg)/xylazine (8 mg/kg) and exposed to PBS (40 μl) or GC frass (40 μg/40 μl) via intratracheal instillation [12]. Mice were given a lethal dose of sodium pentobarbital 24 h later and bronchoalveolar lavage (BAL) fluid was isolated (Figure 1A). These studies were approved by the Cincinnati Children's Hospital Medical Center Institutional Animal Care and Use Committee.

### Assessment of airway inflammation

Lungs were lavaged thoroughly with 1 ml of Hanks balanced salt solution (HBSS) without calcium or magnesium. The bronchoalveolar lavage fluid (BAL) was centrifuged (1,800 rpm for 10 min); the supernatant was removed for TNFα analysis by ELISA (R&D Systems, Minneapolis, MN) and immediately stored at -80°C. Total cell numbers were counted on a hemocytometer. Smears of BAL cells prepared with a Cytospin II (Shandon Thermo, Waltham, MA) were stained with Diff-Quick (Thermo Electron Corporation, Pittsburg, PA) solution for differential cell counting.

### Primary mouse bone marrow-derive neutrophils

Bone marrow from femurs and tibias was isolated, rinsed and red blood cells were lysed. Resuspended cells were layered onto a three step Percoll gradient (52%, 64%, 72%) and centrifuged (1,000 rpm for 30 min at RT). The bottom layer (64%-72%) containing neutrophils was collected, counted and plated as previously described [13]. Cells (1 × 10^6^) were cultured in MEM medium supplemented with 10% fetal bovine serum 50 μg/ml streptomycin, 2 U/mL penicillin, and 2 mM L-glutamine for treatment.

### Cell culture

HL-60 promyelocytic leukemia cells (ATCC, Manassas, VA) were cultured in MEM medium and were differentiated in the presence of 1% DMSO for 3 days. Cells were centrifuged, washed and deprived of serum for 6 h. For all cell culture experiments, cells were treated with the following concentrations of GC frass (1 μg/ml), LPS (1 μg/ml; from E. Coli 055:B5, Sigma Chemical Corp, St. Louis, MO) in the presence or absence of zinc gluconate (1 μM; Spectrum Chemicals, New Brunswick, NJ) and the zinc ionophore, pyrithione (10 μM; 2-mercapopyridine N-oxide, Sigma Chemical Corp). In all cases, zinc was added in the presence of pyrithione, except where noted. Lactase dehydrogenase (LDH) cytotoxicity assay kit (US Biological, Marblehead MA) was performed on cell supernatants to determine if zinc gluconate and/or pyrithione treatment resulted in cell death.

### Assessment of TNFα production

Following treatment, cell culture media was clarified by centrifugation (10,000 g × 10 min) and immediately stored at -80°C. The fluid was analyzed for TNFα using an ELISA kit from R&D Systems (Minneapolis, MN).

### Electrophoretic mobility shift assay (EMSA)

HL-60 cells were treated as described for 1 h. Cells were harvested and nuclear proteins isolated as previously described [14]. All nuclear extraction procedures were performed on ice with ice-cold reagents. Protein concentrations were determined by Bradford assay (Bio-Rad, Hercules, CA) and stored at -70°C until use. The probe was labeled with [γ-^32^P]ATP using T4 polynucleotide kinase (Invitrogen, Carlsbad, CA) and purified in MicroBiospin chromatography column. The gel was run using 10 μg of nuclear protein as previously described [14]. An oligonucleotide probe encoding the consensus sequence of NF-κB was purchased from Santa Cruz (Santa Cruz, CA). Non-radioactive specific and non-specific probes were added at 5× the concentration of the radiolabeled probe. Gels were transferred to Whatman 3 M paper, dried under a vacuum at 80°C for 1 h, and exposed using a Phosphoimager.

### IKK phosphorylation

HL-60 cells were treated as described above for 1 h. Cellular lysate was harvested, and IKK was immunoprecipitated using an IKKγ antibody bound to protein G beads. The beads were pulled down and incubated with GST-IκBα and ^32^P-ATP. The samples were isolated by gel electrophoresis and exposed to film. An IKKγ (Santa Cruz, Santa Cruz, CA) Western blot was performed to confirm equal immunoprecipitation.

### Western blot analysis

Following treatment, whole cell lysates were isolated and separated on an 8-16% Tris-glycine gel (Invitrogen, Carlsbad, CA). A20 (eBioscience, San Diego, CA), RIP1 (R&D Systems, Minneapolis, MN), TRAF6 and β-actin (both from Santa Cruz Biotechnology, Inc., Santa Cruz, CA) were detected by Western Blot analysis.

### Murine model of allergic airway inflammation

For airway administration of allergen, mice were given three intratracheal inhalation challenges of PBS (40 μl) or GC frass (40 μg/40 μl) on days 0, 7, and 14 (n = 6 mice/group). On days 14, 15, and 16, mice were injected i.p. with either PBS or zinc gluconate (10 mg/kg). Mice were harvested on day 17 for allergic airway responses (Figure 1B).

### Airway hyperresponsiveness measurements

Allergen-induced airway hyperresponsiveness (AHR) was determined as we have previously described [15]. Briefly, mice were anesthetized 72 hours after the last GC frass exposure, intubated and ventilated at a rate of 120 breaths per minute with a constant tidal volume of air (0.2 ml), and paralyzed with decamethonium bromide (25 mg/kg). After establishment of a stable airway pressure, 25 μg/kg weight of acetylcholine was injected i.v. and dynamic airway pressure (airway pressure time index [APTI] in cm-H_2_O × sec^-1^) was followed for 5 minutes.

### Serum IgE

Animals were bled and serum isolated for total IgE levels. Serum was also analyzed for zinc levels using the QuantiChrom Zinc Assay kit (BioAssay Systems, Hayward, CA).

### Cytokine production

Liberase/DNase I digests of the lung were prepared to obtain single lung cell suspensions. Single cell suspensions (2.5 × 10^5^) were incubated for 72 hours in culture medium (RPMI) or in RPMI treated with Conconavalin A (10 μg/ml) and supernatants were analyzed by ELISA for cytokine expression as previously described [12].

### Statistical analysis

When applicable, statistical significance was assessed by one-way analysis of variance (ANOVA). Differences identified by ANOVA were pinpointed by Student-Newman-Keuls' multiple range test.

## Results

### Zinc supplementation reduced GC frass-induced airway inflammation

**Figure 2:**
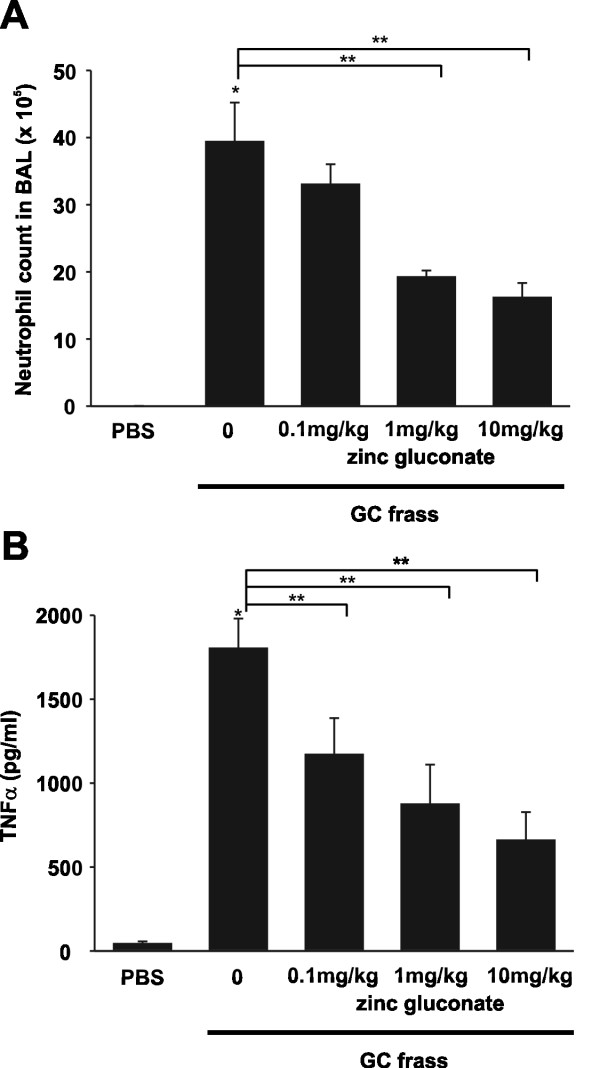
**Zinc gluconate decreased airway inflammation *in vivo***. Naïve BALB/c mice were given an i.p. injection of PBS or zinc gluconate (0.1, 1, or 10 mg/kg) once a day for 3 days prior to a single i.t. inhalation of GC frass (40 μg/40 μl). 18 h later, BAL fluid was harvested, infiltrated cells counted and BAL fluid was analyzed by ELISA. In all cases means ± SEM are shown (n = 6 mice per group). A. Total cells in BAL fluid (different from PBS *p < 0.001; different from GC frass treated **p < 0.05). B. TNFα ELISA (different from PBS * < 0.001; different from GC frass treated without zinc supplementation **p < 0.05, as determined by ANOVA).

**Figure 3:**
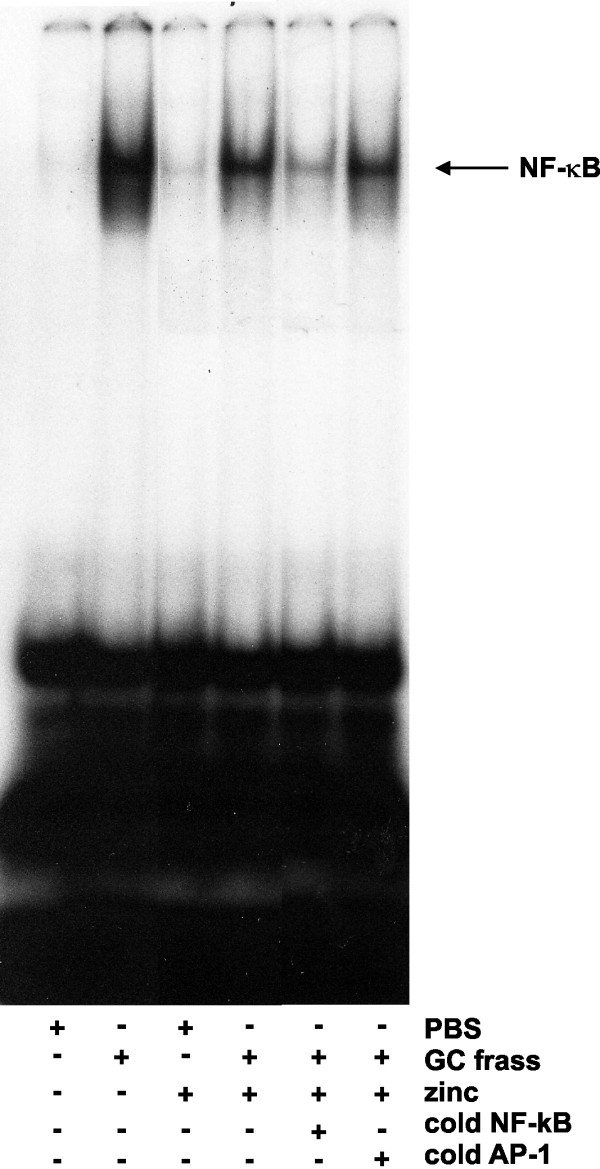
**Zinc supplementation decreased NF-κB-DNA binding in whole lung**. Mice were given daily i.p. injections of PBS or zinc gluconate (10 mg/kg) for 3 days prior to a single i.t. inhalation of PBS or GC frass (40 μg/40 μl). 4 h later, whole lungs were removed and snap frozen. Lungs were homogenized, nuclear extracts isolated and analyzed by EMSA. NF-κB-DNA binding is shown, top arrows designate supershift of p65 and p50. Data shown are representative from a single mouse (n = 3 mice per group).

We previously showed that a single intratracheal inhalation of GC frass to naïve BALB/c mice induced a significant immune response within 18 hours [13]. To determine if zinc supplementation could alter this response, we performed an i.p. injection of increasing doses of zinc gluconate (0.1-10 mg/kg) for 3 days prior to a single intratracheal instillation of GC frass (Figure 1A). As expected, a single exposure to GC frass induced neutrophil infiltration into the BAL fluid (Figure 2A) and increased the inflammatory mediator TNFα (Figure 2B). Importantly, administration of zinc gluconate resulted in decreased neutrophil infiltration into the airways of mice with a concentration of 1 mg/kg, while TNFα secretion was significantly decreased with a dose of 0.1 mg/kg zinc gluconate. Next, we wanted to determine if zinc altered NF-κB activation. To do this, we pretreated mice with zinc gluconate (1 mg/kg) i.p. for 3 days prior to inhalation of GC frass (Figure 1A). 18 h later, lungs were isolated and nuclear extracts were isolated. We confirmed that GC frass increased NF-κB-DNA binding as determined by electrophoretic mobility shift assay (EMSA) in whole lung (Figure 3). Pretreatment with zinc gluconate significantly decreased the level of NF-κB-DNA binding. The specificity of NF-κB was shown by the competition of the radiolabeled band with excess non-labeled NF-κB oligonucleotide and the lack of competition using the non-specific non-labeled AP-1 oligonucleotide. These data confirm that zinc gluconate supplementation can attenuate the inflammatory response by altering NF-κB activation *in vivo*.

### Zinc directly affects neutrophil cytokine production via NF-κB

**Figure 4:**
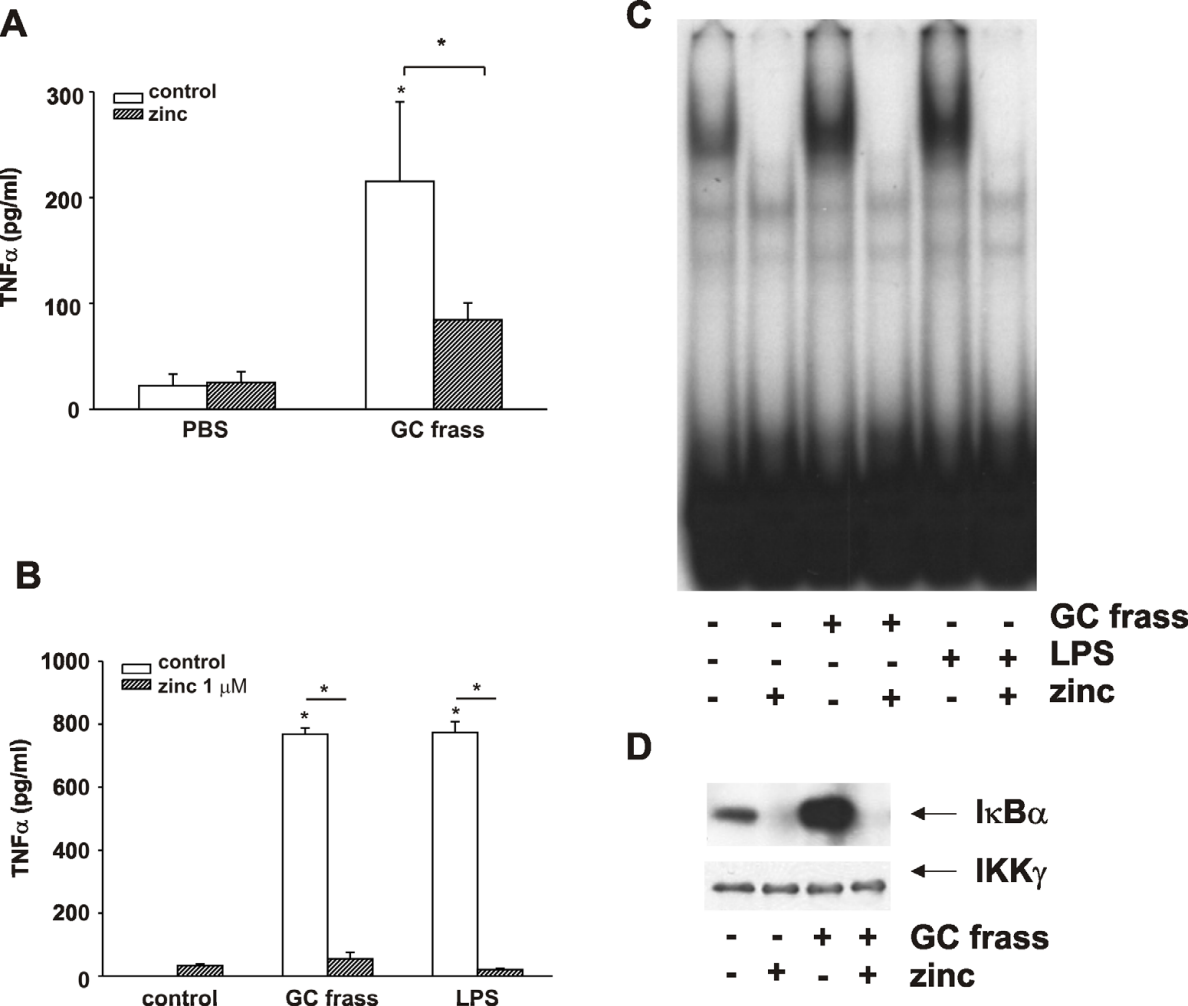
**Zinc supplementation decreased neutrophil-derived cytokine production via decreased NF-κB activation**. A. Bone marrow-derived neutrophils were isolated and treated with GC frass (1 μg/ml) or LPS (1 μg/ml) with or without zinc gluconate (1 μM) and pyrithione (10 μM) for 18 h. Supernatants were clarified and analyzed by ELISA for TNFα production. Data are means ± SEM (n = 3 separate experiments) and statistical differences assessed by ANOVA (*p < 0.05). B. HL-60 cells were treated as in A and analyzed by ELISA for TNFα production. Data are expressed as mean ± SEM for four experiments (*p < 0.001). C. Zinc supplementation decreased NF-κB-DNA binding. HL-60 cells were stimulated with GC frass or LPS in the presence of zinc and pyrithione for 4 h. Nuclear extracts were analyzed for NF-κB-DNA binding by EMSA. D. Zinc supplementation decreased IKK activity. HL-60 cells were cultured with or without zinc gluconate and pyrithione prior to treatment with GC frass for 1 h. IKK activation was assessed by *in vitro *kinase assay using recombinant IκBα as a substrate. PBS treated (P) and GC frass treated (F) cells cultured with and without zinc.

Using the neutrophil as a marker of an early innate immune response, we asked whether zinc supplementation would attenuate an inflammatory response in neutrophils. To do this, we isolated bone marrow-derived neutrophils from naïve wild type mice and treated the cells *ex vivo *with GC frass. We found that GC frass significantly increased TNFα production from bone marrow-derived neutrophils (Figure 4A). Treatment with zinc gluconate (1 μM) in the presence of the zinc ionophore pyrithione (10 μM) significantly reduced GC frass-induced TNFα production. Due to the difficulties involved in obtaining primary neutrophils, we decided to perform additional experiments using HL-60 cells (a human promyelocytic leukemia cell line that becomes neutrophil-like following differentiation with DMSO). GC frass-induced TNFα production in differentiated HL-60 cells was completely abolished in the presence of zinc gluconate/pyrithione (Figure 4B). Since GC frass contains endotoxin [13], an agonist of TLR4, we were also interested in determining if zinc supplementation could alter LPS-induced activation of neutrophils. We confirmed that addition of zinc gluconate was sufficient to completely attenuate LPS-induced cytokine expression (Figure 4B). It is important to note that neither zinc gluconate nor pyrithione, either alone or in combination, resulted in cell death as measured by LDH release (data not shown).

To explore the mechanism by which zinc gluconate regulated TNFα production, we asked whether zinc gluconate could inhibit NF-κB activation and DNA binding. HL-60 cells were differentiated in DMSO for 3 days prior to treatment with GC frass or LPS in the absence or presence of zinc gluconate and pyrithione. As expected, both GC frass and LPS increased NF-κB-DNA binding (Figure 4C). Importantly, NF-κB-DNA binding was completely attenuated with zinc supplementation. Next, we performed a time course of GC frass and LPS treatment in the absence and presence of zinc supplementation and determined the levels of IκBα. We found increased degradation of IκBα following treatment with both GC frass and LPS (data not shown). Zinc supplementation resulted in the sustained presence of IκBα, suggesting less degradation of the protein. Since IκBα phosphorylation and thus degradation is mediated by IKK activation, we asked whether zinc gluconate regulated IKK activity as determined by an *in vitro *kinase assay. GC frass-induced activation of IKK was attenuated in the presence of zinc gluconate (Figure 4D). Together these data suggest a role for zinc gluconate inhibition of NF-κB upstream of IKK activation.

### Regulation of A20 by zinc gluconate

**Figure 5:**
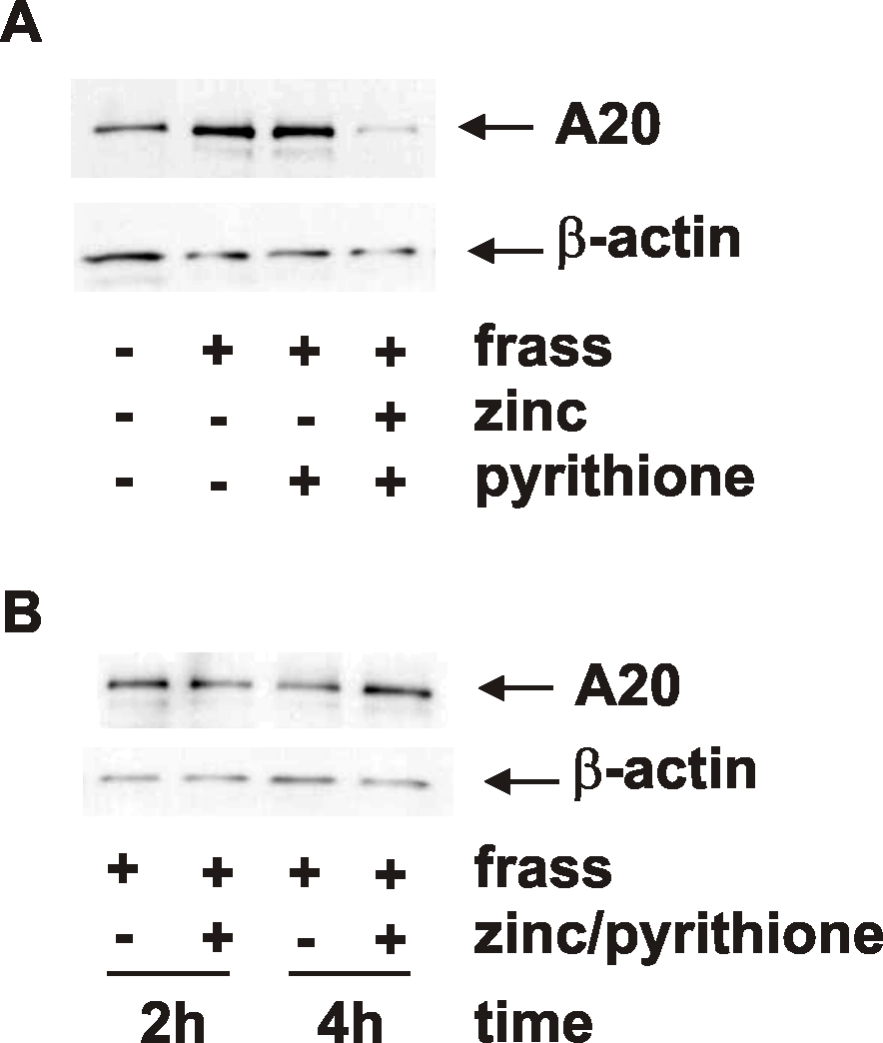
**A20 protein levels following GC frass with or without zinc supplementation**. HL-60 cells were treated with GC frass (1 μg/ml) or LPS (1 μg/ml) with or without zinc gluconate (1 μM) and pyrithione (10 μM) for 18 h (A) or for either 2 or 4 h (B). Cell lysates were harvested, separated by gel electrophoresis and probed with an antibody against A20 or β-actin. Each experiment was performed 3 times.

Given that A20, a seven-zinc finger protein is known to inhibit NF-κB activation, and zinc tempers inflammation, we sought to determine if zinc affects A20 protein levels. We treated differentiated HL-60 cells with GC frass in the presence or absence of zinc gluconate and/or pyrithione and harvested cells 18 h later. We found an upregulation of A20 protein levels following GC frass treatment, which was significantly decreased when zinc/pyrithione were also present (Figure 5A). These results were somewhat expected since we have shown that zinc supplementation resulted in decreased NF-κB activation (refer to Figure 3C) and A20 is a downstream gene product of NF-κB. These data also confirm that pyrithione alone did not alter A20 protein levels. We next determined the stability of A20 protein levels shortly following zinc treatment. To do this, we treated cells with GC frass, zinc and pyrithione and harvested cell lysate at 2 and 4 hours following treatment. We found that zinc did not alter A20 protein levels rapidly following treatment (Figure 5B), suggesting that the decrease in A20 seen at 18 h was most likely due to transcriptional regulation.

### Zinc alters the activity of A20

**Figure 6:**
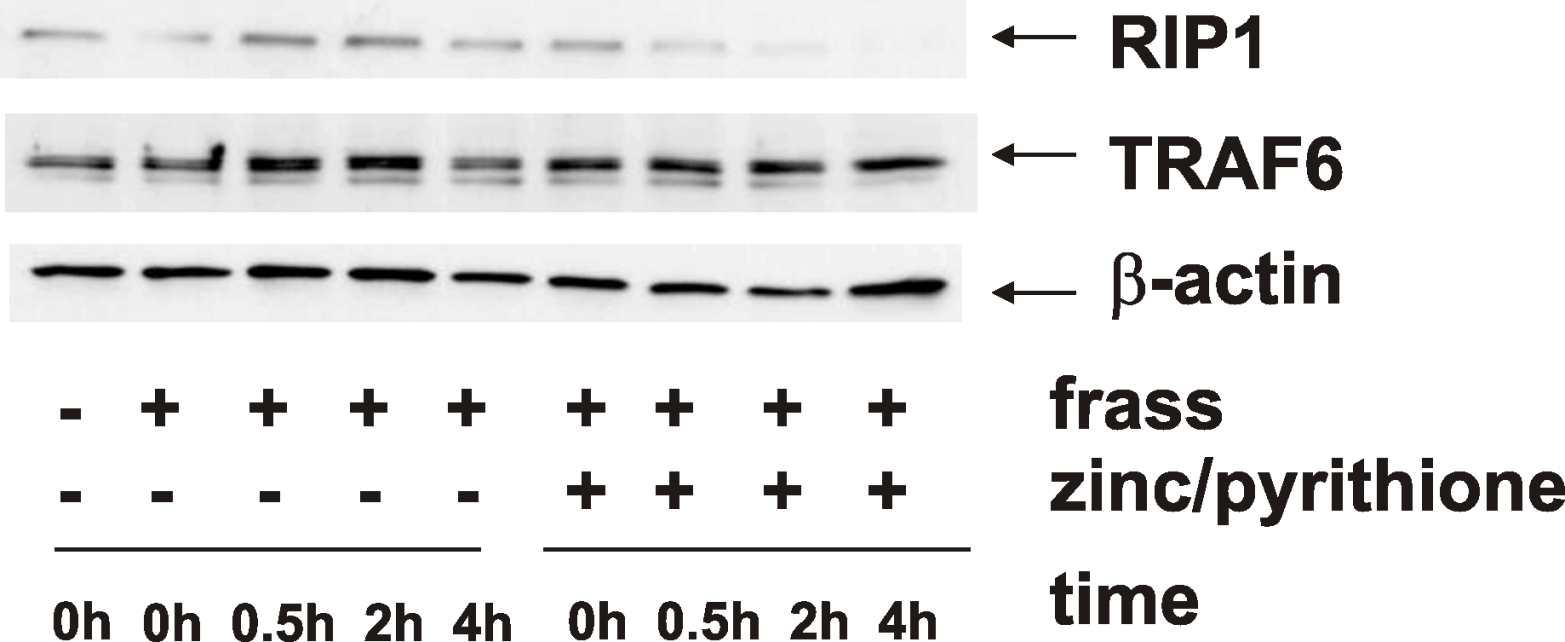
**RIP1 and TRAF6 protein levels following GC frass with or without zinc supplementation**. HL-60 cells were treated with GC frass (1 μg/ml) or LPS (1 μg/ml) with or without zinc gluconate (1 μM) and pyrithione (10 μM) for times as indicated (0-4 hr). Cell lysates were harvested, separated by gel electrophoresis and probed with antibodies against RIP1, TRAF6 or β-actin. This experiment was performed 3 times.

We considered the possibility that zinc gluconate could alter A20 activity to potentially make it more efficient. Since it is known that A20 alters ubiquitination patterns of RIP1 leading to its degradation, we investigated RIP1 protein levels following zinc treatment. To do this, we treated DMSO-differentiated HL-60 cells with GC frass in the presence or absence of zinc and pyrithione and harvested the cells 30 min to 4 h later. Importantly, we found that zinc supplementation resulted in decreased levels of RIP1 following GC frass treatment (Figure 6). In addition, we investigated the levels of TRAF6, another downstream target of A20. We found no changes in the protein levels of TRAF6 following zinc treatment. Together, this data indicates that the addition of zinc resulted in decreased RIP1 protein levels, presumably by altering the ubiquitination state of RIP1 thus causing its degradation. TRAF6 levels remained unaltered; however it is unclear as to whether A20 altered the levels of ubiquitin on TRAF6, or if the ubiquitination state of TRAF6 does not play a role in its degradation.

### Zinc supplementation decreased allergic airway inflammation in mice

**Figure 7:**
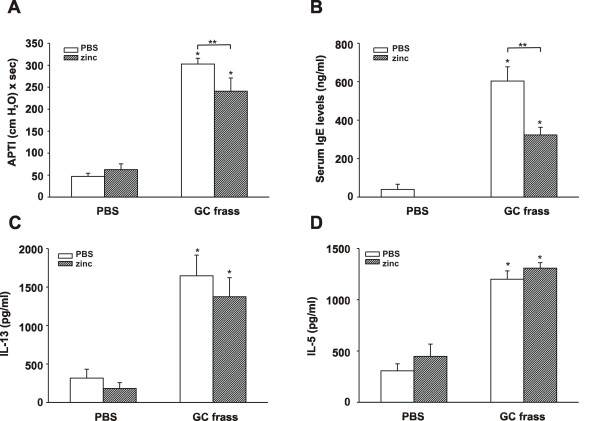
**Zinc supplementation decreased experimentally-induced allergic asthma in mice**. BALB/c mice were sensitized on days 0, 7, and 14 with an intratracheal inhalation of PBS or GC frass (40 μg/40 μl). On day 14, 15, and 16 selected mice were administered i.p. injections of zinc gluconate (10 mg/kg) or PBS. On day 17, mice were anesthetized and acetylcholine was injected after establishment of a stable airway pressure. Blood was collected and BAL fluid was harvested. Lungs were isolated, and cultured for 3 days in the presence of ConA for cytokine analysis by ELISA. In all cases, means ± SEM (n = 6 mice per group) were reported and statistical significance was determined by one-way ANOVA. A. AHR was measured as airway pressure time index (APTI) in cm-H_2_O × sec -1 (compared to PBS *p > 0.001; compared to GC frass **p = 0.034). B. Serum IgE levels (compared to PBS *p > 0.001; compared to GC frass **p > 0.001). C. IL-13 levels (compared to PBS; *p > 0.001). D. IL-5 levels (compared to PBS; *p > 0.001).

Our data indicate that zinc supplementation results in decreased inflammation due to decreased NF-κB activity. We were very interested in determining if zinc supplementation could alter experimentally-induced allergic airway inflammation in our established mouse model. To do this, we first induced an airway response by sensitizing and challenging mice to GC frass on days 0, 7, and 14. We have previously shown this method to induce significant airway hyperresponsiveness to acetylcholine, increased serum IgE, increased eosinophilia and increased Th2 (IL-4, IL-5 and IL-13) cytokine expression [16,17]. We then administered zinc gluconate or PBS i.p. to mice on days 14, 15, and 16. On day 17, mice were analyzed for allergic airway inflammation (refer to Figure 1B). Serum zinc levels rose in the zinc gluconate-treated mice from a baseline of 9.05 ± 0.6 μM to 11.8 ± 0.5 μM (n = 12; p = 0.002). As expected, sensitization and challenge to GC frass resulted in increased airway responsiveness to cholinergic agent (Figure 7A), increased serum IgE levels (Figure 7B), and increased Th2 cytokine expression (Figure 7C and 7D). Interestingly, the addition of zinc gluconate into the i.p. cavity of mice following sensitization and challenge was able to significantly decrease airway hyperresponsiveness and serum IgE levels. There was no significant effect of zinc gluconate on the Th2 cytokines IL-13 or IL-5 or IL-4 (data not shown). In addition, administration of zinc gluconate following allergen sensitization and challenge resulted in decreased airway eosinophil numbers (3315 ± 519 cells following allergen challenge compared to 2593 ± 587 cells with zinc supplementation), although these levels did not reach statistical significance. In our asthma model, airway neutrophilia is also present following allergen sensitization and challenge (2639 ± 813) and was decreased following administration of zinc gluconate (1094 ± 268; n = 6 mice per group; p < 0.05). Together these data suggest that supplementation with high doses of zinc gluconate alters some aspects of experimentally-induced allergic airway inflammation in a mouse model.

## Discussion

In this report, we describe a role for zinc supplementation in modulating the innate immune response to a common allergen *in vivo *which is characterized by cellular infiltration and cytokine release into the airways of mice. In addition, we found that zinc supplementation is sufficient to decrease airway hyperresponsiveness and serum IgE levels suggesting an important therapeutic treatment. Our data suggests that zinc works to temper inflammation at the level of A20 within the NF-κB pathway. A20 is a zinc-fingered protein that is a well-known inhibitor of the NF-κB pathway and was thus a good potential target for the location of zinc's anti-inflammatory effect within this pathway. In our study, we found that zinc supplementation, when added concurrently with GC frass, did not affect A20 levels in the short term (4 h), but resulted in decreased A20 protein levels at 18 h. We postulate that this was most likely due to transcriptional regulation with the enhancement of the NF-κB negative feedback loop and inhibition of continued activation of NF-κB, thus eventually decreasing A20 protein production. It has been shown that zinc can induce increased A20 levels in the presence of a variety of stimuli including LPS [18] and phorbol myristate acetate (PMA) [19]. These experiments differed from ours in that media containing zinc was added to cells for 10 days prior to stimulation, but they support our interpretation that zinc regulates A20 to alter NF-κB activity.

Our results indicate a potential mechanism by which zinc regulates NF-κB activity; i.e. by enhancing the activity of A20 thus resulting in de-ubiquitination of RIP1 and leading to its degradation. The ubiquitin editing abilities of A20 were recently examined and better defined. Shembade et. al. [5] showed that A20 removes the activating chains of ubiquitin from locus K63 on RIP1 and facilitates the addition of inhibitory chains to locus K48, targeting RIP1 for degradation and thus preventing the activation of IKK downstream. They also showed that A20 inhibits the polyubiquitinization and activation of TRAF6, a process that is dependent on Ubc13 enzymatic activity. Ubc13 is also ubiquitinated at K48 by A20 and targeted for degradation, but this occurs late after stimulation. TRAF6 is then rendered inactive, though not degraded [5]. Utilizing these findings, we were able to evaluate A20 activity using RIP1 and TRAF6 protein levels as a surrogate. With respect to A20 activity, we were able to show that RIP1 was degraded more quickly in the presence of zinc gluconate after stimulation with GC frass, but TRAF6 levels remained unchanged. These findings suggest that the addition of zinc gluconate augments the activity of A20; enabling it to act more rapidly on RIP1 resulting in its degradation and thus ceasing the NF-κB inflammatory cascade more quickly. A limitation of the current study was our evaluation of A20 activity via RIP1 as an indirect assessment; however our studies clearly show that in the presence of zinc, RIP1 degradation is enhanced, suggesting an increased proteosomal targeting due to altered ubiquitin states. Our findings suggest that zinc is acting on the NF-κB pathway at the level of A20 to further enhance its inhibitory effects.

In this study we also wanted to investigate zinc as an anti-inflammatory with potential therapeutic utility for airway inflammation. Asthma is a chronic inflammatory disease of the airways and is characterized by increased mucus production and airway hyperresponsiveness. Several studies have shown a role for increased NF-κB in asthmatics, including increased NF-κB p65 protein abundance, IκBα phosphorylation and IKK activity in peripheral blood mononuclear cells (PBMC) of uncontrolled asthmatics compared to normal individuals [20] and greater levels of NF-κB p65 and p50 activation in cultured bronchial epithelial cells from untreated asthmatics than controls [21]. NF-κB was also found to be upregulated in bronchial epithelial cells a murine model of ovalbumin-induced allergic airway inflammation [22]. The importance of NF-κB signaling in allergic inflammation and mucus production was shown using mice with IKK-deficient Clara epithelial cells [23]. In this study, we found a significant reduction in airway hyperresponsiveness in our murine model by administering zinc gluconate for three days following the final allergen exposure. Importantly, the levels of serum IgE were substantially decreased following zinc administration. IgE is known to bind to specific Fcε receptors on mast cells thus result in release of histamine and other inflammatory substances to produce an allergic cascade. Zinc supplementation did not appear to have any effect on levels of the Th2 cytokines IL-13, IL-5 and IL-4. Much of the literature on zinc and asthma has focused primarily on levels of dietary zinc; however Lang et. al. [24] recently showed that zinc supplementation following establishment of allergic inflammation reduced the levels of eosinophils and lymphocytes in the BAL fluid of mice. They failed to find a significant difference in the amount of mucus hyperplasia, determined by PAS staining, following zinc administration however. We also found decreased numbers of eosinophils and neutrophils in the BAL fluid of mice; however unlike neutrophil levels, the decrease in eosinophil levels did not reach statistical significance. It is possible that the amount of times zinc is administered (i.e. 3 times in our protocol compared to twice a week from days 34-52 in the Lang protocol) was responsible for the different results. To our knowledge, this is the first report showing that zinc supplementation can modulate airway responsiveness to cholinergic agents.

The literature on zinc as a potent antioxidant is vast but what role this may have in airway inflammation remains unclear. Recent studies have looked at abnormal distributions of trace minerals, including zinc, as an instigator of oxidative damage and inflammation in asthma [25,26]. Prasad et. al. [18] investigated the role of zinc as an antioxidant and after supplementation to human subjects, noted a decrease in oxidative stress and NF-κB activation in isolated mononuclear cells when compared to placebo. TNFα and IL-1β are inflammatory cytokines known to exert oxidative stress on cells via the generation of reactive oxygen species (ROS). In their study, they also noted a decrease in TNFα and IL-1β cytokine levels in their volunteers who took zinc supplements, suggesting that one way in which zinc functions as an antioxidant is by negatively regulating the gene expression of these inflammatory cytokines to prevent the formation of ROS [18]. Thus, zinc's role as an antioxidant likely works concurrently with its anti-inflammatory role via similar or overlapping biochemical mechanisms. Similar to Prasad et. al., we were able to show that zinc negatively inhibits NF-κB-induced cytokine production. We acknowledge that some of the positive effects noted on airway hyperresponsivity and inflammation may have been an antioxidant effect of zinc supplementation. However, we continue to promote the supplementation of zinc as a therapeutic to temper the inflammation as well as the oxidative stress induced by allergic stimuli in the airways.

## Conclusion

Zinc gluconate is an inexpensive medication, readily available and administered both parenterally and orally. With the severity and prevalence of asthma on the rise, our data suggest the possibility of a readily available, easily dosed, and cost effective manner to aid in the suppression of airway hyperresponsiveness.

## List of abbreviations

AHR: airway hyperresponsiveness; BAL: bronchoalveolar lavage; GC: German cockroach; IκBα: I kappa B alpha; IKK: I kappa kinase; i.p.: intraperitoneal; LPS: lipopolysaccharide; NF-κB: nuclear factor kappa B; RIP1: receptor-interacting protein 1; ROS: reactive oxygen species; TNFα: tumor necrosis factor alpha; TNFR: tumor necrosis factor receptor; TLR: toll-like receptor; TRAF6: TNF receptor-associated factor 6.

## Competing interests

The authors declare that they have no competing interests.

## Authors' contributions

CIM participated in the design and implementation of the experiments and drafted the manuscript. JRL performed the animal experiments, ELISAs and Western blots. PZ performed the cell culture work, EMSAs and in vitro kinase assays. KP conceived of the study, participated in its design and coordination and drafted the manuscript. All authors read and approved the final manuscript.
